# Seroprevalence of transfusion transmitted infections among different blood group donors at Blood Bank LUMHS, Hyderabad

**DOI:** 10.12669/pjms.332.11691

**Published:** 2017

**Authors:** Faheem Ahmed Memon, Ikram Din Ujjan, Amir Iqbal Memon, Abdul Rehman Shaikh, Ali Raza Rao, Arshi Naz

**Affiliations:** 1 Dr. Faheem Ahmed Memon, MBBS. Liaquat University of Medical and Health Sciences, Jamshoro, Sindh, Pakistan; 2 Prof. Ikram Din Ujjan, MBBS, PhD. Liaquat University of Medical and Health Sciences, Jamshoro, Sindh, Pakistan; 3 Amir Iqbal Memon, MBBS. Liaquat University of Medical and Health Sciences, Jamshoro, Sindh, Pakistan; 4 Dr. Abdul Rehman Shaikh, MBBS. Liaquat University of Medical and Health Sciences, Jamshoro, Sindh, Pakistan; 5 Ali Raza Rao, BS. Diagnostic and Research Laboratory, Hyderabad, Pakistan. Liaquat University of Medical and Health Sciences, Jamshoro, Sindh, Pakistan; 6 Dr. Arshi Naz, PhD. National Institute of Blood Diseases and Bone Marrow Transplantation, Karachi, Pakistan

**Keywords:** HCV, HBV, HIV, Transfusion, Syphilis, Transfusion Transmitted Infections

## Abstract

**Objectives:**

To study the prevalence of HBsAg, Anti-HCV, HIV, Syphilis and Malaria in blood donors.

**Methods:**

This is a cross sectional descriptive study, conducted at Blood bank and Transfusion center at Liaquat University of Medical & Health Sciences (LUMHS) Hyderabad, during the period from January, 2014 to June, 2015. A total of 4683 blood donors were screened for HBsAg, Anti-HCV and HIV on Architect 20001 (manufactured by Abbott), employing chemiluminescent microparticle immunoassay (CMIA). For Syphilis, VDRL ICT kits were used and Malaria parasite was screen through MP slides. Blood grouping was performed by both forward and reverse methods.

**Results:**

This study showed a high frequency of HBsAg, VDRL and malaria positivity among the O-ve blood group donors, i.e. 3.70%, 9.25% and 0.61% respectively. Blood group B-ve individuals were commonly infected with HCV (12.5%) as compared with all other blood group donors. HIV is more commonly reported in A+ve blood group individuals. Blood group O+ve is more prevalent (37.41 %).

**Conclusion:**

High frequency of HCV infection in blood donors advocates implementation of strict screening policy for donors and public awareness campaigns about preventive measures to reduce the spread of this infection as well as other transfusion transmissible infections.

## INTRODUCTION

Blood is the major source of transmission of infectious diseases like hepatitis C virus (HCV), human immunodeficiency virus (HIV), hepatitis B virus (HBV), syphilis, malaria, and many other infections. To prevent transmission of these infections screening is carried out routinely in all blood transfusion centers.^1^ Screening methods for the infectious diseases especially Carbonyl-metallo-immunoassay (CMEIA) and Nucleic Acid testing (NAT) have excellent sensitivity and specificity and helps to enhance the safety of the blood transfusion, reducing the diagnostic window period as much as possible.

Liver disease due to HBV has become an enormous problem globally.^2^ It is estimated that worldwide two billion people have been infected with HBV and more than 350 million have chronic lifelong infection.^3^ It is estimated that 170 million people are chronically infected with HCV and that more than three million are newly infected each year.^4^

Overall frequency of HCV infection in general population of Pakistan ranges from 4-25% as shown by different studies.^5^ According to a WHO global survey conducted in 2006, 39.5 million individuals were found to have been infected with HIV 1 and 2.^6^ In another WHO report,36 million people are found to be infected with Treponema palladium.^7^

Recently ABO blood groups were studied not only for transfusion science but also its association with various diseases e.g. malaria, salivary gland tumors, colorectal cancer, carcinoma of stomach, thyroid disorders, ovarian tumors and small cell lung cancer.^8^ A report showed a link between ABO blood type and pancreatic cancer i.e. a higher association of pancreatic cancer with the non-O blood groups, compared to O.9 However, these findings to date are inconsistent, and the exact mechanism linking blood groups and its association with infectious diseases is currently unclear; the concept, therefore, needs further investigation.

The objective of this study was to determine the frequency of HBsAg, HCV, HIV, Syphilis and Malaria in various ABO and Rh (D) blood groups donors.

## METHODS

This cross sectional and descriptive study was conducted at Blood Bank and Transfusion center LUMHS Hyderabad during the period from January 2014 to June 2015, after approval from institutional ethical committee. A total of 4683 blood donors fulfilling the donor selecting criteria were included. Two blood samples were collected from each donor. Ethylene diamine tetra acetic acid (EDTA) sample was collected for blood grouping and malaria screening while clotted sample for screening of HBsAg, Anti-HCV, HIV and syphilis. HBsAg, Anti-HCV and HIV were analyzed by CMIA on Architect 2000I (Abbott Diagnostics^®^). Sample Cutoff values for HIV Ag/Ab Combo, HbsAg Qualitative II and Anti HCV were documented < 1.00 as non reactive. Specificity and Sensitivity of individual viral markers were mentioned in kit literature as HIV ≥ 99.5% /100%, HbsAg > 99.5% / 100% and HCV 99.6% / 99.1%. Syphilis was tested by immune chromatographic device with ABON Biopharm (Hangzhou) kit while malaria was screened through thick and thin smear using Romanowsky stain. Blood grouping [ABO and Rh (D)] were performed by cell and serum type on tube method. Data were analyzed using basic tools by MS Excel.

**Fig.1:**
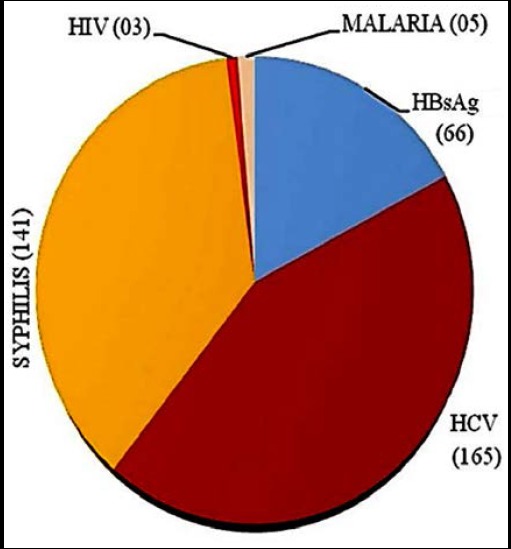
Prevalence of transfusion transmitted infections in the blood donors (N=468).

## RESULTS

Among 4683 blood donors, 1038 were A+ve. Of these, 15 were HBsAg+ve, 42 were anti HCV+ve, 30 were VDRL+ve for syphilis, one each was HIV and malaria positive. Among the 54 A-ve donors three were anti-HCV+ve. A total of 1275 individuals had B+ve blood group. Among these, 18 were HBsAg+ve, 54 were anti-HCV +ve, 39 were VDRL+ve, 01 each was +ve for HIV and malaria. A total of 96 donors were B-ve; with 3 HBsAg+ve, 12 Anti HCV+ve and 03 positive for VDRL(syphilis). The most prevalent blood type was O+ve (n=1752). Among these, 15 were HBsAg +ve, 45 were Anti HCV +ve, 45 were VDRL+ve for syphilis, one each was positive for HIV and malaria. O-ve blood group individuals were 162 in strength among whom 06 were HBsAg +ve, 03 were Anti HCV +ve, 15 were VDRL +ve for syphilis, 01 was +ve for malaria. AB+ve were 279 from which 09 were HBsAg +ve, 06 were anti-HCV +ve, 09 were VDRL +ve for syphilis, 01 was +ve for malaria. None of the AB-ve donors (n=27) tested positive for the screened infections.

This study showed a high frequency of HBsAg, VDRL and malaria positivity among the O-ve blood group donors, i.e. 3.70%, 9.25% and 0.61% respectively. Blood group B-ve individuals were more infected with HCV (12.5%) as compare to all other blood group donors. HIV is more commonly reported in A+ve blood group individual (Table-I).

**Table-I:** Frequency of viral markers including malaria and syphilis in different types of blood groups of healthy donors.

*Blood group*	*No. of donors N (%)*	*Hbs AgN (%)*	*Anti HCVN (%)*	*SyphilisN (%)*	*HIVN (%)*	*MalariaN (%)*
A +ve	1038(22.16)	15(1.45)	42(4.04)	30(2.89)	01(0.09)	01(0.09)
A -ve	54(01.15)	00(0.00)	03(5.55)	00(0.00)	00(0.00)	00(0.00)
B +ve	1275(27.22)	18(1.41)	54(4.23)	39(3.05)	01(0.07)	01(0.07)
B -ve	96(02.10)	03(3.12)	12(12.5)	03(3.15)	00(0.00)	00(0.00)
O +ve	1752(37.41)	15(0.85)	45(2.56)	45(2.56)	01(0.05)	01(0.05)
O -ve	162(03.45)	06(3.70)	03(1.85)	15(9.25)	00(0.00)	01(0.61)
AB +ve	279(05.95)	09(3.22)	06(2.15)	09(3.22)	00(0.00)	01(0.35)
AB -ve	27(00.57)	00(0.00)	00(0.00)	00(0.00)	00(0.00)	00(0.00)
	4683	66(1.40)	165(3.52)	141(3.01)	03(0.06)	05(0.10)

Frequency of Blood groups and transfusion transmitted infections were expressed in numbers and in percentage % in parenthesis.

## DISCUSSION

Blood and blood components are the only source for transfusion therapy. Recently, advanced screening techniques are utilized to ensure safe blood transfusion. Although donors were physically examined and fulfilling the history form, seropositive case was detected. This study was conducted not only to determine the seropositivity among blood donors but also its association with various ABO blood groups. Till date only a few such studies have been conducted in Pakistan.

Blood group O+ve (37.41%) were the most common blood group among the blood group donors. This result is consistent with that of Bhatti and Sheikh (1999)^9^ while inconsistent with Khaskheli and Qureshi (1994).^10^ ABO blood groups studies across Pakistan mostly acknowledge that B+ve is the most prevalent blood group.^11^,^12^ Variations in phenotypic expression of ABO blood group may be due to geographical distribution, extended tribes and ethnic groups in local population.

Since blood group O+ve is more common in Pakistani population so it seems that infections are also prevalent in this group of blood donors (37.41%). Infectious disease like HbsAg and syphilis were noted to be more common in O-ve blood group, i.e. 3.70% and 9.25% respectively. HCV were highly prevalent in B-ve blood group(12.5%). Saeed and Mujtaba^1^(2011) reported a high seropositivity of HbsAg and HCV in O+ve blood group. The prevalence of HbsAg and HCV may vary due the niche of disease.

Higher incidence of syphilis (9.25%) and malaria (0.61%) were noted among O-ve blood group subjects while HIV (0.09%) were mostly prevalent in A+ve blood group individuals (Table-I). In 2012 Sobia and Sanaullah et al. reported that the prevalence of HIV in blood donors was 0% (1992), 0.0086% (2002) 0.06% (2011).^13^ Limited data exist for HIV and syphilis infection in Pakistani blood donors.

ABO blood group antigens play important role not only as receptors or ligands but also in modulation of immune response.^14^ Several investigators have reported an association between ABO blood groups and risk factor for HBV and HCV infections.^15^-^17^ The exact mechanism of association of ABO blood group and mentioned diseases is not clear.

Tyagi et al (2013) reported that there is preference for Rh negative blood group by the TTIs and even the specificity of a particular infection to a particular blood group which is matching in four TTIs in our study.^18^

In our study, the total numbers of ABO-RhD negative donors are low as compared to the ABO- RhD positive blood donors. This is because of naturally low percentage of Rh negative blood group in humans but RhD negative blood donors show higher percentage of sero-positivity for TTIs.^19^

### Limitation of the Study

The major limitation of current study was selection of donor population from a single center.

## CONCLUSION

HBsAg, syphilis and malaria is more common in blood group O-ve, HCV is more common in blood group B-ve and HIV is more common in A+ve blood group donors. Association of TTIs with blood group and RhD types needs random sampling on large scale in general population.

### Authors’ Contribution

**FAM** designed study, statistical analysis & writing of manuscript.

**IDU, AIM** supervised the study and secured funding.

**ARS, ARR** did data collection and helped in manuscript writing.

**AN** did review, final approval of manuscript and is responsible for the clinical integrity of the study.
